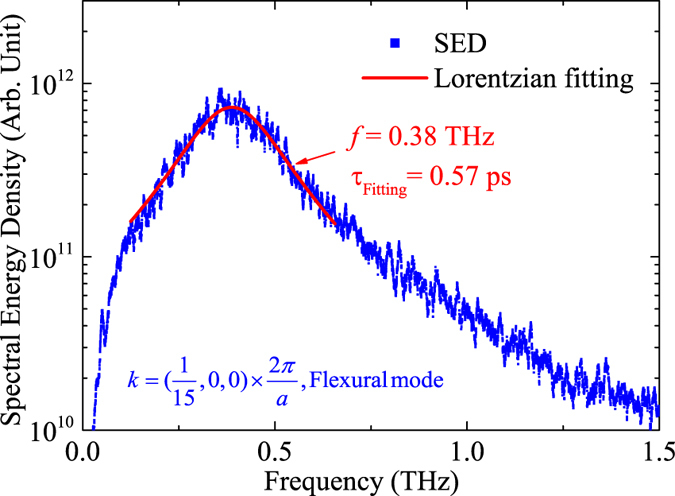# Thermal transport in bismuth telluride quintuple layer: mode-resolved phonon properties and substrate effects

**DOI:** 10.1038/srep27492

**Published:** 2016-06-06

**Authors:** Cheng Shao, Hua Bao

**Affiliations:** 1 University of Michigan-Shanghai Jiao Tong University Joint Institute, Shanghai Jiao Tong University, Shanghai 200240, China

## Abstract

The successful exfoliation of atomically-thin bismuth telluride (Bi_2_Te_3_) quintuple layer (QL) attracts tremendous research interest in this strongly anharmonic quasi-two-dimensional material. The thermal transport properties of this material are not well understood, especially the mode-wise properties and when it is coupled with a substrate. In this work, we have performed molecular dynamics simulations and normal mode analysis to study the mode-resolved thermal transport in freestanding and supported Bi_2_Te_3_ QL. The detailed mode-wise phonon properties are calculated and the accumulated thermal conductivities with respect to phonon mean free path (MFP) are constructed. It is shown that 60% of the thermal transport is contributed by phonons with MFP longer than 20 nm. Coupling with *a*-SiO_2_ substrate leads to about 60% reduction of thermal conductivity. Through varying the interfacial coupling strength and the atomic mass of substrate, we also find that phonon in Bi_2_Te_3_ QL is more strongly scattered by interfacial potential and its transport process is less affected by the dynamics of substrate. Our study provides an in-depth understanding of heat transport in Bi_2_Te_3_ QL and is helpful in further tailoring its thermal property through nanostructuring.

Bismuth telluride as one of the best room temperature thermoelectric materials has attracted significant attention recently^1^,^2^,^3^,^4^,^5^. The performance of thermoelectric materials is characterized by its figure of merit


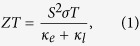


where *S*, σ, *T, κ*_*e*_, and *κ*_*l*_ are the Seebeck coefficient, electrical conductivity, absolute temperature, electrical thermal conductivity, and lattice thermal conductivity, respectively. One effective approach to enhance *ZT* is to reduce the lattice thermal conductivity of a thermoelectric material^1^,^2^,^3^. Significant progress has been made in the last decade to decrease the lattice thermal conductivity of Bi_2_Te_3_-based materials. For example, in nanostructured bismuth antimony telluride bulk alloy, a peak *ZT* of 1.4 can be achieved at 100 °C^1^. The *ZT* value of p-type Bi_2_Te_3_/Sb_2_Te_3_ superlattice was reported to be as high as 2.4 at room temperature, which is mainly due to the controlled transport of phonons and electrons in the superlattices^2^. Comparing to nanostructured bulk materials, low dimensional materials have large surface-to-volume ratios and size effects, which could be utilized to further enhance the *ZT* value^6^,^7^,^8^. A few quintuple layers (QLs) Bi_2_Te_3_ recently attracted great interest on its thermoelectric property due to its quasi-two-dimensional (2D) structure^9^. For example, Zhang *et al*. studied the electron and phonon transport properties in Bi_2_Te_3_ QL and found that the *ZT* value can be optimized to as high as 2.2 at 800 K^10^. Qiu and Ruan investigated the thermal transport in few-QLs Bi_2_Te_3_ thin films using molecular dynamics (MD) simulations^11^. They found that the thermal conductivity of Bi_2_Te_3_ QL first decreases and then increases with the number of QLs, which is attributed to the interplay between Umklapp scattering and boundary scattering^11^. It is also shown that the thermal conductivity of QLs can be reduced by introducing nanopores^11^. More recently, Park *et al*. calculated the thermal conductivity of Bi_2_Te_3_ thin films by solving the Boltzmann transport equation under the relaxation time approximation^12^. They extended the Klemens-Callaway’s model to incorporate the phonon scattering process due to the van der Waals force at the interface of adjacent Bi_2_Te_3_ QLs and could correctly reproduce the anisotropic heat transport of Bi_2_Te_3_^12^. While these studies provided a better understanding of electronic and thermal transport properties in Bi_2_Te_3_ QL, detailed information about mode-resolved phonon transport properties and its contribution to thermal conductivity is still missing. Such information is crucial for a better understanding of thermal transport and tailoring the thermal conductivity^13^,^14^.

On the other hand, in the experimental characterization of thermal properties, the 2D Bi_2_Te_3_ QLs could be supported on a substrate^15^,^16^. More generally, the coupling with another material (typically unavoidable) could affect the thermal transport of low-dimensional materials^5^,^17^. There have been quite a few studies focusing on the thermal transport of low-dimensional materials when it is weakly bonded to another material (typically to a substrate)^18^,^19^,^20^,^21^. It is generally believed that the properties of low dimensional materials are very sensitive to the presence of substrate^18^,^19^,^20^,^21^. For example, Ong *et al*. studied the substrate effects on thermal transport in graphene and found that coupling to the substrate can reduce the thermal conductivity of graphene by an order of magnitude, but further increasing the coupling strength will enhance the thermal transport, due to the coupling of flexural acoustic phonons with substrate Rayleigh waves^19^. Qiu and Ruan performed a phonon spectral analysis to study the effect of silicon dioxide substrate on graphene^22^. They found that the presence of silicon dioxide substrate can reduce the phonon relaxation times (RTs) on all phonon branches^22^. There are also some studies showing that the thermal conductivity of could be enhanced due to the coupling with another material. For example, Zhang *et al*. studied the effect of substrate on heat transport in two-dimensional (2D) silicene and found that depending on the different crystal types of silicon carbide substrate, the thermal conductivity of 2D silicene could be either enhanced or suppressed^23^. Guo *et al*. investigated thermal transport in two coupled Fermi-Pasta-Ulam chains. The results show that in certain regions the coupling to another chain can increase the thermal conductivity of the chain^20^. They believe that the enhancement of thermal conductivity is due to the reduction of anharmonic phonon scattering, which is induced by the shift of the phonon band to the low wave vector^20^. Bi_2_Te_3_ QL is different from those materials in previous studies, because it has strong anharmonicity and extremely low thermal conductivity. The detailed effects of substrate on its thermal transport remain unclear and a systematic investigation is highly desirable.

In this work, we adopt MD based methods to study the phonon transport in 2D Bi_2_Te_3_ QL. Thermal conductivities of freestanding and supported Bi_2_Te_3_ QL are first calculated through non-equilibrium molecular dynamics (NEMD) and Green-Kubo methods. Then the normal mode analysis (NMA) method is employed to obtain the mode-resolved phonon transport properties in Bi_2_Te_3_ QL. The structure of Bi_2_Te_3_ QL coupled with an *a*-SiO_2_ substrate is also investigated to explore the substrate effects. Accumulated thermal conductivities with respect to MFP for both freestanding and supported Bi_2_Te_3_ are constructed to quantify the contribution of different phonon modes to thermal conductivity. We also investigated the effect of coupling strength and the atomic mass of substrate on the heat transfer in Bi_2_Te_3_ QL.

## Methods

### Molecular dynamics simulations

In this work, MD based methods are adopted to study the thermal transport properties of Bi_2_Te_3_ QL. LAMMPS package is used to carry out all the MD simulations^24^. It should be noted that there are also some recent works using the first-principles methods to study the thermal properties in Bi_2_Te_3_^10^,^25^. Although first-principles simulation can extract more accurate force constants, it is still difficult to incorporate higher order anharmonicity^26^,^27^. The large computational cost also made it is difficult to be applied to study a Bi_2_Te_3_ QL or the effect of the external perturbation from a substrate.

**Figure 1:**
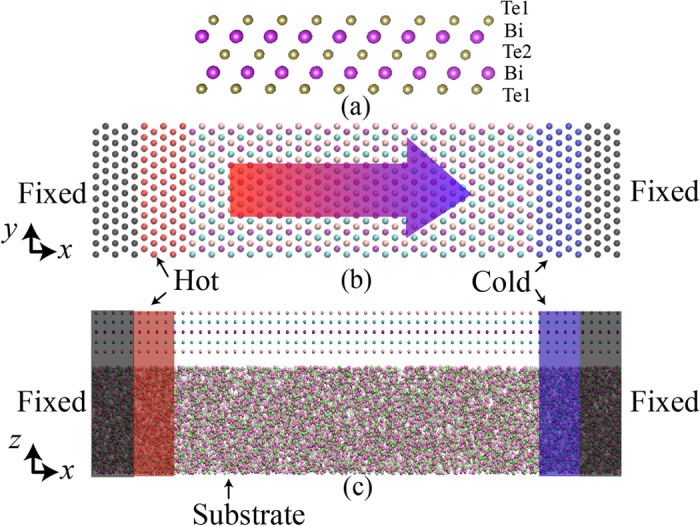
(**a**) Atomic structure (side view) of Bi_2_Te_3_ QL. (**b**) NEMD simulation model (top view) for freestanding Bi_2_Te_3_. (**c**) NEMD simulation model (side view) for supported Bi_2_Te_3_.

The atomic structure of Bi_2_Te_3_ QL is shown in Fig. 1(a). It contains five atomic layers with a sequence of Te1-Bi-Te2-Bi-Te1. Each layer has a hexagonal crystal structure with lattice constant *a* = 4.369 Å. The thickness of the QL is 10.14 Å, which is one-third of lattice constant of bulk Bi_2_Te_3_ along the cross-plane direction. A two-body Morse-type potential developed by Qiu *et al*. is used to model the interaction between atoms in Bi_2_Te_3_ QL^28^. An *a*-SiO_2_ substrate is used to study the effect of substrate on heat transport in Bi_2_Te_3_ QL. We choose *a*-SiO_2_ for its widely used in experiments^15^. A Tersoff-type potential parameterized by Munetoh *et al*.^29^ is used to model the interaction between Si and O atoms. To generate the *a*-SiO_2_, we first heat the *β*-cristobalite to 6000 K using a Langevin thermostat and maintain that temperature for 10 ps. Then the system is slowly quenched to 300 K at a rate of 10^12^ K/s^30^. The *a*-SiO_2_ substrate is 2 nm thick, which should be thick enough to eliminate the size effect based on the previous studies^22^,^31^,^32^. Because of the van der Waals nature of interaction between the Bi_2_Te_3_ QL and substrate, a Lennard-Jones (LJ) potential is adopted to describe the interaction, which has the form of


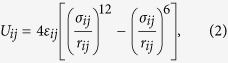


where ε_*ij*_ is the potential well depth, *σ*_*ij*_ is the distance at which the potential energy reaches zero, and *r*_*ij*_ is the separation between atoms *i* and *j*. In this study, the parameters are obtained based on the universal force model^33^, which gives ε_Bi-Si_ = 17.9 meV, *σ*_Bi-Si_ = 3.9 Å, ε_Bi-O_ = 7.6 meV, *σ*_Bi-O_ = 3.5 Å, ε_Te-Si_ = 17.3 meV, *σ*_Te-Si_ = 3.9 Å, ε_Te-O_ = 6.7 meV, and *σ*_Te-O_ = 3.5 Å. The cutoff distance for LJ potential is set to be 10 Å.

We use both NEMD and Green-Kubo methods to calculate the thermal conductivity of freestanding Bi_2_Te_3_ QL, and use NEMD to calculate the thermal conductivity of supported Bi_2_Te_3_ QL. The schematic of the simulation domain used in NEMD is shown in Fig. 1(b,c). The outermost regions are static regions in which the atoms are fixed during the NEMD process. Next to the fixed regions are the hot and cold thermal reservoirs. Periodic boundary condition is applied to all three directions, while a vacuum layer of 5 nm is used in *z* direction (cross plane direction) to model the freestanding situation. The width (*y* direction) of the simulation domain is set to be 6 nm after convergence tests. It is well known that NEMD methods suffer from the finite size effect in predicting the thermal conductivity. In order to eliminate this effect, we vary the domain size along the *x* direction and use the extrapolation method to obtain the thermal conductivity. In freestanding cases, we vary the length from 16 to 80 nm. For the supported cases, due to the large computational costs, the maximum length along the heat flux direction is 40 nm. A time step of 2 fs is used the simulations of freestanding Bi_2_Te_3_ QL. Due to the existence high of frequency vibration modes in *a*-SiO_2_ substrate, we use a time step of 0.25 fs for supported Bi_2_Te_3_ QL. The system is first equilibrated in an NVT ensemble at 300 K for 0.5 ns, and then switched to NPT ensemble to release the internal stress for 1 ns. After that atoms at two ends are fixed and the temperature of two reservoirs are maintained at 320 K and 280 K respectively through the velocity rescaling method^34^. After obtaining the steady state temperature gradient and heat flux across the system, Fourier’s law is applied to calculate the thermal conductivity.

For the Green-Kubo method, the thermal conductivity is obtained from the autocorrelation of the heat current vector. Equilibrium molecular dynamic (EMD) simulations are carried out to calculate heat current vectors. The simulation domain used for EMD simulations is 5.6 × 5.2 nm^2^ in the *x* and *y* direction (containing 910 atoms), while in *z* direction a length of 10 nm is used to model the freestanding structure. According to the previous MD study on Bi_2_Te_3_ QL^11^, our simulation domain size is large enough to obtain converged thermal conductivity. We first run the pre-equilibrium procedures similar to the NEMD simulation to make sure the system reaches equilibrium state. After that the system is switched to an NVE ensemble for 2 ns and the heat current data is recorded every 10 fs. These heat current data are then used to calculate the heat current autocorrelation function (HCACF). Fifteen independent simulations (different in initial velocities) are used to explore the phase space and make sure our results are true ensemble average^30^,^35^. Here a direct integral method is used to integrate the HCACF and to obtain the thermal conductivity. It should be noted that in some previous works, a low-pass filter is used to remove the optical component in the HCACF and the exponential fitting method is used to extract the final thermal conductivity^28^,^36^. To make a comparison, we used the directed integral method to integrate the HCACF with and without a low-pass filter (the cutoff frequency is 0.5 THz) and found they yield similar results.

### Normal mode analysis

While NEMD and Green-Kubo method can provide an overall thermal conductivity, no information at the phonon level can be obtained. In order to obtain better insights about heat transport in Bi_2_Te_3_ QL, a mode-wise analysis is necessary. In normal mode analysis (NMA), the trajectories of each atom are projected onto the normal modes and the amplitude of each normal mode can be written as^37^,^38^


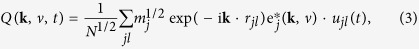


where *Q*(**k**, *ν, t*) denotes the amplitude of a normal-mode with wave vector **k** and polarization *ν* at time *t, N* is the number of unit cells in the simulation domain, *m* is the mass, *r* is the equilibrium position of an atom, e(**k**, *ν*) is the mode eigenvector, and *u* is the displacement. The subscript *j* denotes the *j*-th atom in a unit cell, *l* denotes the *l*-th unit cell and the superscript ^*^denotes the complex conjugate.

When the lattice is purely harmonic, the normal-mode amplitudes will be periodic functions of time. The anharmonic forces cause the damping of the normal-mode. If the anharmonic effect is small, the normal-mode amplitudes can be written as^39^


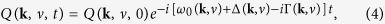


where *ω*_0_(**k**, *ν*) is the unperturbed harmonic frequency of this mode, Δ(**k**, *ν*) is the frequency shift, and Γ(**k**, *ν*) is the linewidth. The phonon spectral linewidth Γ(**k**, *ν*) and the phonon relaxation time *τ*(**k**, *ν*) have the following relationship


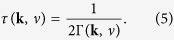


In order to obtain the phonon relaxation time, we apply a Fourier transform to the time derivative of each normal-mode and express the spectral energy density (SED) of this mode as:


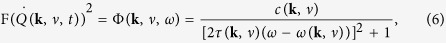


where 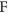 denotes the Fourier transform, 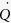
(**k**, *ν, t*) is the time derivative of *Q*(**k**, *ν, t*), *ω*(**k**, *ν*) is the anharmonic phonon frequency (equals to *ω*_0_(**k**, *ν*) + Δ(**k**, *ν*) from Equation (4)), and Φ(**k**, *ν*,*ω*) is the SED of this mode and can be expressed as a Lorentzian function. The phonon frequency and relaxation time are obtained by fitting the SED plot with a Lorentzian function. The location of peak gives the phonon frequency and the inverse of full width at half maximum 2Γ is the phonon relaxation time *τ*.

Similar to previous studies, we make the isotropic approximation for the first Brillouin zone^32^,^40^. A simulation domain of 13.1 × 6.1 nm^2^ in the *x* and *y* direction is used in our study. It should be noted that available k points are depending on the size of simulation domain^41^, so there are 21 k points available along the Γ to K direction in our simulations. The lattice dynamics program GULP is used to calculate the eigenvectors^42^. The atomic positions and velocities are recorded at a 10 fs interval during an NVE process at 300 K. A trajectory of 20 ns is used in the NMA to extract the phonon frequencies and relaxation times. We also apply the NMA to bulk Bi_2_Te_3_. In bulk Bi_2_Te_3_ the lattice constant along the cross-plane direction is 30.42 Å, which contains three QLs. Periodic boundary condition is used in the cross-plane direction to model the bulk situation. All the other simulation details are similar to that used for Bi_2_Te_3_ QL.

## Results and Discussions

### Thermal conductivity of freestanding and supported Bi_2_Te_3_ QL

**Figure 2:**
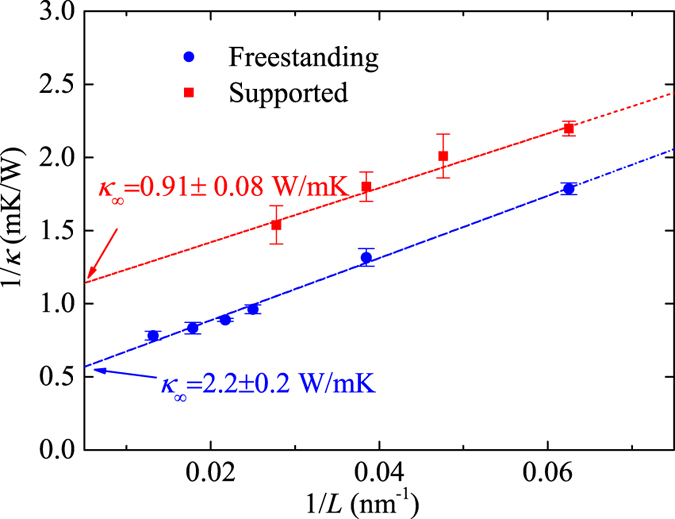


**Figure 3:**
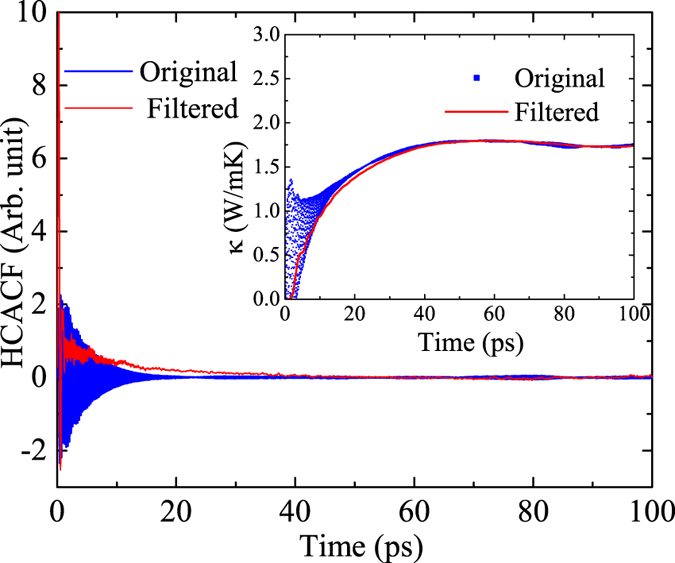
Typical in-plane HCACF in freestanding Bi_2_Te_3_ QL before and after a low-pass filter (the cutoff frequency is 0.5 THz). Inset: The direct integral of HCACFs.

Thermal conductivity values of freestanding and supported Bi_2_Te_3_ QL at different lengths calculated from the NEMD method are shown in Fig. 2. These data points were extrapolated with a linear function to obtain the thermal conductivity at infinite length^43^, which are 2.2 ± 0.2 W/mK and 0.91 ± 0.08 W/mK for freestanding and supported cases, respectively. For the freestanding case, there seems to be an onset of a convergence of the thermal conductivity at the point when *L* = 80 nm (1/*L* = 0.0125 nm^−1^). Adopting linear fitting may overestimate the thermal conductivity. Therefore, we also tried to extrapolate thermal conductivity using a quadratic fitting, and it yields 2.1 W/mK. The Green-Kubo method was also used to calculate the in-plane thermal conductivity of freestanding Bi_2_Te_3_ QL for comparison. To obtain the thermal conductivity, one can either fit the HCACF with exponential function, or directly integrate the HCACF. In a previous work by Qiu and Ruan^28^, the former approach was adopted and a low-pass filter was used to remove the high frequency oscillation. In our simulation the direct integration method is used. From Fig. 3, one can see that whether removing the high frequency oscillation components of the HCACF does not obviously affect the final thermal conductivity value. The result is 1.8 ± 0.1 W/mK, which is consistent with our NEMD result and agrees well with the work by Qiu and Ruan (1.7 ± 0.4 W/mK)^11^. To compare with experiment, Teweldebrhan *et al*.^15^ measured the thermal conductivity of stacked few QLs and found that the thermal conductivity is about 1.1 W/mK, which is slightly lower than our simulation results.

Supporting on an *a*-SiO_2_ substrate leads to a ∼60% reduction in thermal conductivity based on our NEMD simulation results. Such a magnitude of reduction is smaller compared to some other 2D materials that supported on the same substrate. For example, Ong *et al*. found that the thermal conductivity of graphene is reduced by an order of magnitude once supported on an *a*-SiO_2_ substrate^19^. Wang *et al*. also reported a 78% reduction in thermal conductivity of silicene supported on *a*-SiO_2_ substrate^32^. Comparing to these monolayer 2D materials, the substrate has less effect on the thermal conductivity of Bi_2_Te_3_ QL. This is likely due to the five-layer structure of Bi_2_Te_3_ QL: only the lowest layer is strongly coupled with the substrate while the other layers are relatively weakly coupled. In another work that studies the supported multilayer graphene, it was also found that the substrate suppression of the thermal conductivity is less prominent in multilayer graphene^44^.

### Mode-wise phonon properties in Bi_2_Te_3_ QL

**Figure 4:**
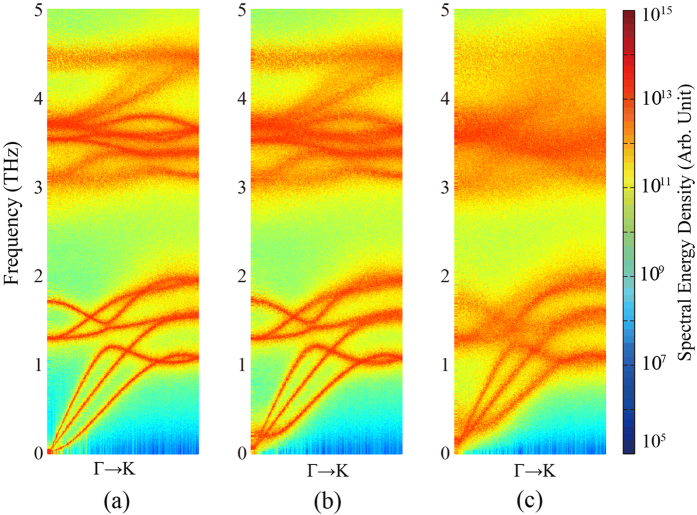
Spectral energy density of (**a**) freestanding Bi_2_Te_3_ QL, (**b**) supported Bi_2_Te_3_ QL, and (**c**) supported Bi_2_Te_3_ QL with coupling strength 5 times of the origin one.

In order to gain a better understanding of thermal conductivity reduction in the supported Bi_2_Te_3_ QL, we perform the NMA to the freestanding and supported QL. The SED spectra of freestanding and supported QL are firstly calculated for comparison. In order to obtain a better resolution in the SED figures, a larger simulation domain (131 × 6.1 nm^2^ in the *x* and *y* direction) was adopted so that 201 k-points can be extracted along Γ to K direction. The total simulation time is reduced to 2 ns. The phonon SEDs at points along the Γ to K direction of freestanding and supported Bi_2_Te_3_ QL are shown in Fig. 4(a,b). The SED spectra of supported Bi_2_Te_3_ with a stronger coupling strength (5 times of the original one) are also shown in Fig. 4(c). The magnitude of the SED is represented by different colors, as shown in Fig. 4. The location of local maximum in the SED spectra gives the frequency of a normal mode (or phonon mode) while the broadening of the local maximum gives the phonon linewidth. Therefore, the local peaks in SED spectra give the phonon dispersion curve and the span of the peaks represents the phonon RTs.

A major difference between the supported and freestanding QL is the zone center flexural modes. For supported case, the flexural mode near the zone center is flattened and significantly broadened. A similar flattening of ZA-mode was also reported in a previous study of few layer graphene supported on *a*-SiO_2_ substrate^45^,^46^. From their studies, the coupling to the substrate has twofold effects on the supported graphene^45^,^46^. First, the dispersion of the flexural mode upward shifted from the origin. Second, the flexural mode will become linearized due to the hybridization with the surface Rayleigh mode^45^,^46^. For the supported Bi_2_Te_3_ QL, the flexural mode indeed shifted upward but the linearization is not clearly observed. The dispersion curve in supported Bi_2_Te_3_ QL became blurred comparing to the freestanding QL, which indicates a relatively shorter phonon RTs in the supported case. From Fig. 4(c) it can be seen that the dispersion curve became further blurred with the increasing of coupling strength. This indicates that the substrate could introduce additional phonon scattering, and the scattering strength increases with the increasing of coupling strength.

**Figure 5:**
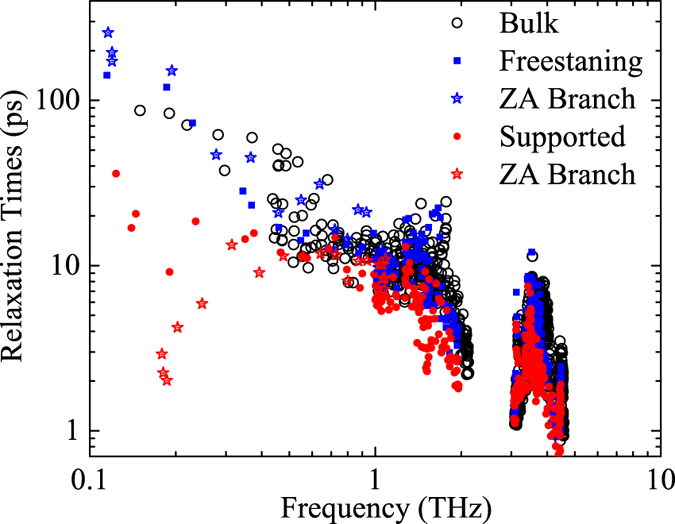
Phonon relaxation times in freestanding and supported Bi_2_Te_3_ QL at 300 K. The relaxation times in bulk Bi_2_Te_3_ are also shown in figure for comparison.

To quantitatively evaluate the effect of substrate on supported Bi_2_Te_3_ QL, we calculate the phonon RTs by fitting the SED spectra with a Lorentz function as described in Eq. (6). The results are shown in Fig. 5. Phonon RTs along Γ to K point in bulk Bi_2_Te_3_ are also shown for comparison. It can be seen that the RTs in freestanding Bi_2_Te_3_ QL can be as long as 200 ps in the low frequency region. Supporting on a substrate leads to a reduction in RTs within the frequency range. The reduction of RTs is especially strong for flexural modes, which are marked by stars in Fig. 5. The average RTs of flexural modes in freestanding QL is 46 ps, while in the supported case the average RTs is reduced to 8.5 ps. It is also reported in graphene that the flexural modes are more sensitive to the presence of substrate^19^,^22^. We believe there are two major reasons for the strong reduction of ZA mode RTs. First, the potential field from the substrate can break the selection rule for ZA phonon scattering, so that a three phonon process involving odd number of ZA modes could occur in the supported QL. This gives a larger scattering rate of ZA modes^47^. Second, the ZA modes in QL will hybridize with the substrate Rayleigh mode, which leads to an enhanced scattering rate between the ZA mode and the substrate mode^46^.

### Thermal conductivity analysis

After obtaining the spectral phonon properties from the NMA and making the assumption that the first Brillouin zone is isotropic, we can express the thermal conductivity as^32^,^40^


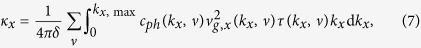


where *κ*_*x*_ is the thermal conductivity in *x* direction, *δ* is the thickness of Bi_2_Te_3_ QL, ν represents different polarization, *c*_*ph*_(*k*_*x*_, *ν*) is the phonon specific heat of phonon mode (*k*_*x*_, *ν*), *v*_*g*,*x*_(*k*_*x*_, *ν*) is the phonon group velocity in *x* direction calculated from harmonic lattice dynamics, and *τ*(*k*_*x*_, *ν*) is the phonon relaxation time.

The overall thermal conductivities of freestanding and supported Bi_2_Te_3_ QL predicted by NMA are 2.1 and 1.0 W/mK, which agree relativity well with that calculated from NEMD and Green-Kubo method. From the NMA calculation, the substrate leads to a ~50% reduction in thermal conductivity, which is also similar to the NEMD results.

**Figure 6:**
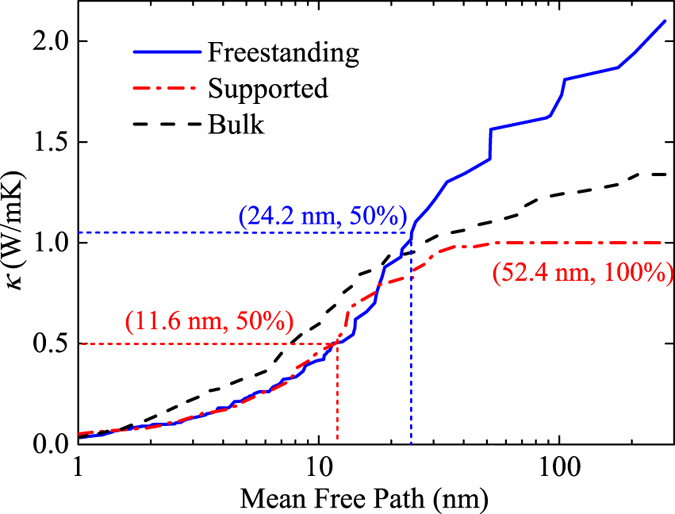
Accumulated thermal conductivities in freestanding and supported Bi_2_Te_3_ QL with respect to phonon MFP. The accumulated thermal conductivity in bulk Bi_2_Te_3_ is also shown figure for comparison.

We further build the accumulated thermal conductivity with respect to MFP, which can be expressed as


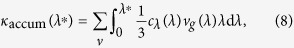


where λ^*^ is the cutoff MFP, *c*_*λ*_(*λ*) is the volumetric heat capacity per unit MFP, and the sum is over all polarizations. The phonon MFP λ and relaxation time *τ* has the relationship *λ* = *v*_*g*_*τ*. In practice, we used a discrete summation over phonon modes with MFP less than λ^*^ to calculate the integral in Eq. (8). The accumulated thermal conductivities are shown in Fig. 6. Comparing the accumulated thermal conductivity of freestanding and supported Bi_2_Te_3_ QL, it can be seen that the reduction in thermal conductivity of supported QL is mainly due to the suppression of long MFP phonon modes. Below 20 nm, the accumulated thermal conductivity of freestanding and supported Bi_2_Te_3_ QL are almost the same, while the thermal conductivity contributed by phonon with MFPs larger than 20 nm is 1.2 W/mK in freestanding Bi_2_Te_3_ QL and 0.2 W/mK in supported Bi_2_Te_3_ QL. We can also see that half of the heat in freestanding Bi_2_Te_3_ QL is carried by phonons with MFPs larger than 52.4 nm, while in supported QL this value is reduced to 24.2 nm. The accumulated thermal conductivity as a function of MFP for bulk Bi_2_Te_3_ is also shown in Fig. 6 for comparison. It has been previously demonstrated by EMD simulations that Bi_2_Te_3_ QL has a larger thermal conductivity than bulk Bi_2_Te_3_^11^. From the accumulated thermal conductivity function, it can be seen that such an enhancement is mainly contributed by the phonons with MFPs longer than 25 nm (phonon modes near zone center) in Bi_2_Te_3_ QL.

**Figure 7:**
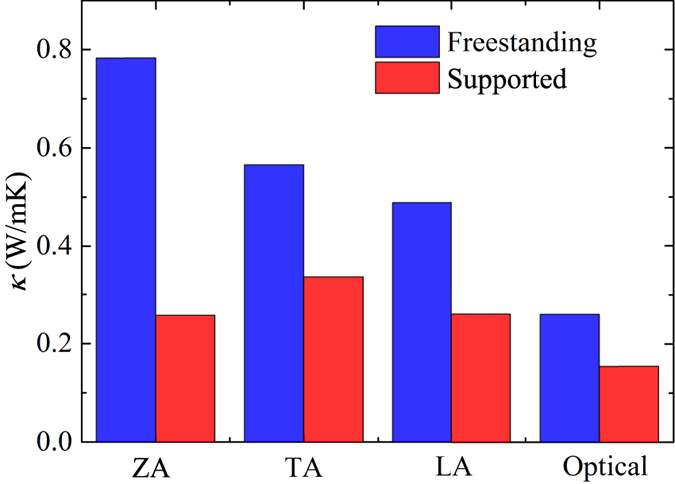


The thermal conductivity contributed from different branches, namely out-of-plane (ZA), longitudinal acoustic (LA), transverse acoustic (TA), and optical branches are also analyzed and the results are shown in Fig. 7. For freestanding Bi_2_Te_3_ QL, the ZA branch contributes most of the thermal conductivity (37%) and the contribution from all three acoustic branches (ZA, TA and LA branch) is 88%. The contribution from optical branch is very small. It should be noted that the large contribution from ZA mode to the heat transfer is not unusual for 2D materials. For materials with reflectional symmetry in the *xy* plane (e.g. graphene), due to the unique selection rule for phonon scattering, the ZA modes tend to scatter less with other phonon modes^47^. Placing on the substrate will suppress the thermal conductivity of all branches, while the suppression in ZA branch is the most significant. From the relaxation times in Fig. 5, it can be seen that this is mainly due to the largely reduced in relaxation time of ZA modes.

### Effect of substrate atomic mass and interaction strength

**Figure 8:**
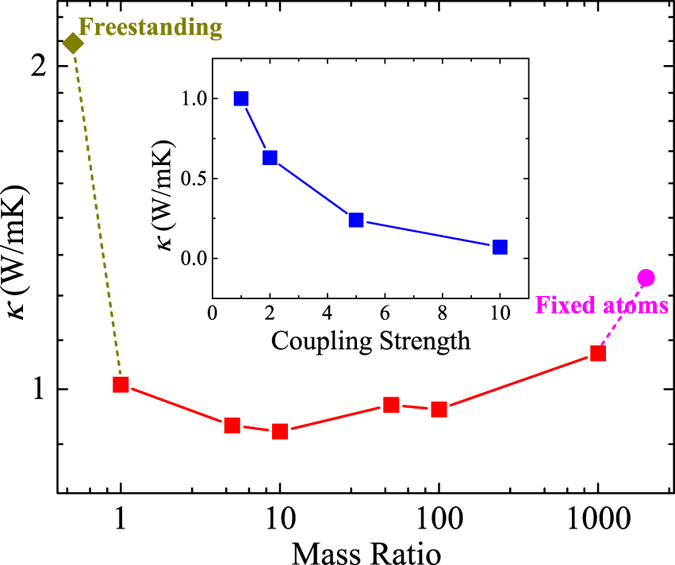


When a 2D material is coupled with another material, for example, supported by a substrate, the detailed interaction and adhesion energy may be quite different, depending on the material types, the interfacial geometry, and the surface polarity of substrate^48^,^49^. It is also reported that the thermal conductivity of supported materials could be quite different depending on the coupling strength and mass ratio to the substrate^20^. In fact this feature could be utilized to tune the thermal conductivity of supported materials^20^,^23^. To explore the effects of different substrate, we generalize our substrate to a virtual one with different atomic masses and coupling strengths with the supported QL. The NMA in combination with Eq. (7) is used to calculate the thermal conductivity of supported Bi_2_Te_3_ QL on different virtual substrates. The coupling strength is tuned by scaling the potential depth ε in the LJ potential for all atom-pairs between the substrate and the QL. The results are shown as the inset of Fig. 8. We can see that increasing the coupling strength tends to further reduce the thermal conductivity of Bi_2_Te_3_ QL. Doubling the interaction strength could reduce the thermal conductivity to 0.63 W/mK. Further increasing the coupling strength leads to a further reduction in thermal conductivity. It should be noted that in the case of 10 times the coupling strength, the adhesion energy between substrate and QL becomes 0.91 J/m^2^, while the adhesion energy between the atomic layers in QL is 9.8 J/m^2^. In this case the QL and the substrate are relatively strongly coupled and it can be inferred from Fig. 4 that the zone center flexural branch is flattened due to the existence of substrate. This means that the phonon frequencies and eigenvectors of the supported QL are changed due to the perturbation from the substrate. In such case, whether the eigenvectors for the freestanding QL are applicable for the perturbed system remains unclear^50^,^51^. To demonstrate the applicability of the unperturbed eigenvectors to the perturbed system, we check the SED profiles of zone center flexural modes with the 10 times coupling strength and plot a typical spectrum (flexural mode with wavevector *k* = (1/15, 0, 0) × 2*π*/*a*) in Fig. 9. We can clearly see a single peak in the SED profile with no significant disruption, indicating that the eigenvectors of the freestanding QL are robust enough to resolve the phonon modes in the supported QL. This further confirms the accuracy of our NMA calculation on the RTs and thermal conductivity for the supported case.

We also scale the atomic mass of all atoms in the substrate by a factor (mass ratio) to explore the effects of substrate atomic mass on the thermal transport in supported materials. The relationship between thermal conductivity and atomic mass ratio of substrate is shown in Fig. 8. We can see that with the increasing of the atomic mass, thermal conductivity of supported Bi_2_Te_3_ QL does not change much, except for the cases when the mass ratio is 1000 or when the atoms fixed. When the atomic mass is 1000, the substrate becomes almost immovable and the direct scattering process between phonon in Bi_2_Te_3_ QL and phonons in substrate is largely suppressed^45^. The presence of substrate can affect the thermal transport in supported materials from two different aspects: the static potential at the interface introduced by the substrate and the dynamics of the substrate itself 52. These two effects can be decoupled through comparing the thermal conductivity of QL under different environment: freestanding, supported, and supported on a substrate with fixed atoms. For the case of substrate with fixed atoms, the reduction of thermal conductivity is mainly due to the static potential at the interface. From Fig. 8, it can be seen that this effect leads to a 40% reduction in thermal conductivity. Allowing the atoms in the substrate to move will introduce additional scattering with substrate phonons, which leads to an additional 13% reduction in thermal conductivity.

### Summary

To summarize, we have systematically investigated the mode-resolved phonon transport properties of freestanding and supported Bi_2_Te_3_ QL by the MD-based NMA methods. The thermal conductivities of freestanding and supported Bi_2_Te_3_ QL are predicted to be 2.1 and 1.0 W/mK, respectively. These values are consistent with the predictions from NEMD and Green-Kubo methods. It is shown that the phonon RTs in the Bi_2_Te_3_ QL range from 1 to 200 ps. Some low frequency phonons have evidently larger relaxation time than that in the bulk Bi_2_Te_3_, which are responsible for the larger thermal conductivity of the QL comparing to bulk phase. The accumulated thermal conductivity is also constructed, which shows that about 60% of the heat is carried by phonons with MFP longer than 20 nm in the QL. The low thermal conductivity of supported QL is mainly attributed to the reduced phonon RTs of all the frequency range, and most significantly for ZA phonon modes. By further varying the coupling strength with substrate and the atomic mass of substrate, we conclude that interfacial scattering is the dominating factor that leads to a reduction of about 40% of the thermal conductivity of Bi_2_Te_3_ QL. Further increasing the coupling strength can significantly suppress the thermal conductivity in Bi_2_Te_3_ QL. Scattering with the substrate phonons can reduce the thermal conductivity by about 13%. Moderate variation of the atomic mass of the substrate has a relatively small effect on the thermal conductivity of the supported QL.

## Additional Information

**How to cite this article**: Shao, C. and Bao, H. Thermal transport in bismuth telluride quintuple layer: mode-resolved phonon properties and substrate effects. *Sci. Rep.* **6**, 27492; doi: 10.1038/srep27492 (2016).

## Figures and Tables

**Figure 9:**